# Laparoscopic resection of a spontaneous gastrocutaneous fistula in an adult – A case report

**DOI:** 10.1016/j.ijscr.2024.110509

**Published:** 2024-10-22

**Authors:** Andrew F. Sabour, Hannah Murawsky, Brian Ward

**Affiliations:** Division of Surgery, Kirk Kerkorian School of Medicine at UNLV, Las Vegas, NV, United States of America

**Keywords:** Case report, Gastrocutaneous fistula, Laparoscopic, Gastrostomy tube

## Abstract

**Introduction:**

Persistent gastrocutaneous fistula (GCF) remains a rare but known complication after gastrostomy tube removal. In children, the gold standard of treatment is surgical through an open fistula takedown. Adults, on the other hand, have a much smaller incidence rate, creating a more difficult dilemma in management.

**Presentation of case:**

We present an unusual case of a 42-year-old male who developed spontaneous opening of his gastrocutaneous fistula decades after its original closure in infancy. Patient initially tried both conservative management and endoscopic suturing but developed recurrence less than a year later. He was subsequently referred to General Surgery and underwent laparoscopic fistula takedown without any complications.

**Discussion:**

Gastrocutaneous fistulas in adulthood may present with various etiologies including distant histories of tube removals.

**Conclusion:**

Surgical management through a minimally invasive technique provides both definitive and efficient care.

## Highlights

• Delayed patency of a gastrocutaneous fistula after gastrostomy tube removal is rare
• Little is known regarding the risk factors of GC fistulas after tube removal in adults
• When medical and endoscopic management fails, surgical treatment remains the gold standard
• An outpatient, laparoscopic approach is a viable option for treatment of patent GC fistulas

## 1 Introduction

Gastrostomy tubes are a commonly used tool in providing direct parenteral nutrition. Once a gastrostomy tube is removed, the leftover tract either spontaneously closes or persists into a chronically patent GCF. The length of time a gastrostomy tube remains correlates to the risk of forming a persistently patent GCF [[1], [2], [3]]. Multiple studies have focused on methods to treat these fistulas, including conservative, endoscopic, and surgical options [1,[4], [5], [6], [7], [8]]. However, certain scenarios exist where a patient with initial closure of their fistula tract then demonstrates patency years later. For these delayed cases, very few studies exist with no description of risk factors or management [9].

In this report, we present an adult with spontaneous patency of his fistula 4 decades after an uncomplicated gastrostomy tube removal. We believe this case holds merit in evaluating the risk factors for delayed GCF patency and offering a minimally invasive approach for definitive therapy. All of the following work has been reported in line with the SCARE criteria [10].

## 2 Presentation of case

A 42-year-old male presented to clinic with a history of intermittent discharge from a left upper quadrant fistula site. Symptoms first began two years ago after a brief hospitalization for COVID pneumonia. Patient stated that his symptoms worsened with any oral intake and described his discharge as liquid mixed with undigested food. Pertinent history included poor oral intake as an infant requiring placement of an open gastrostomy tube at seven days of age. Tube was removed after a total of five months with no complications since.

**Fig. 1:**
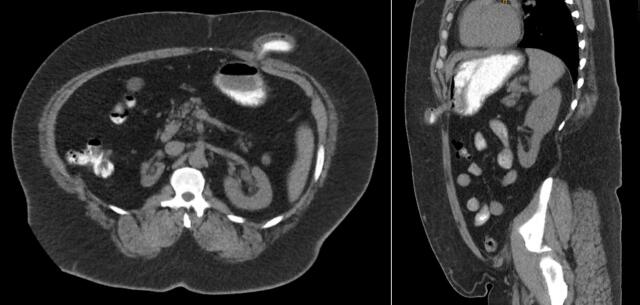
CT abdomen/pelvis scan with oral gastrogaffin contrast demonstrating a patent GCF.

**Fig. 2:**
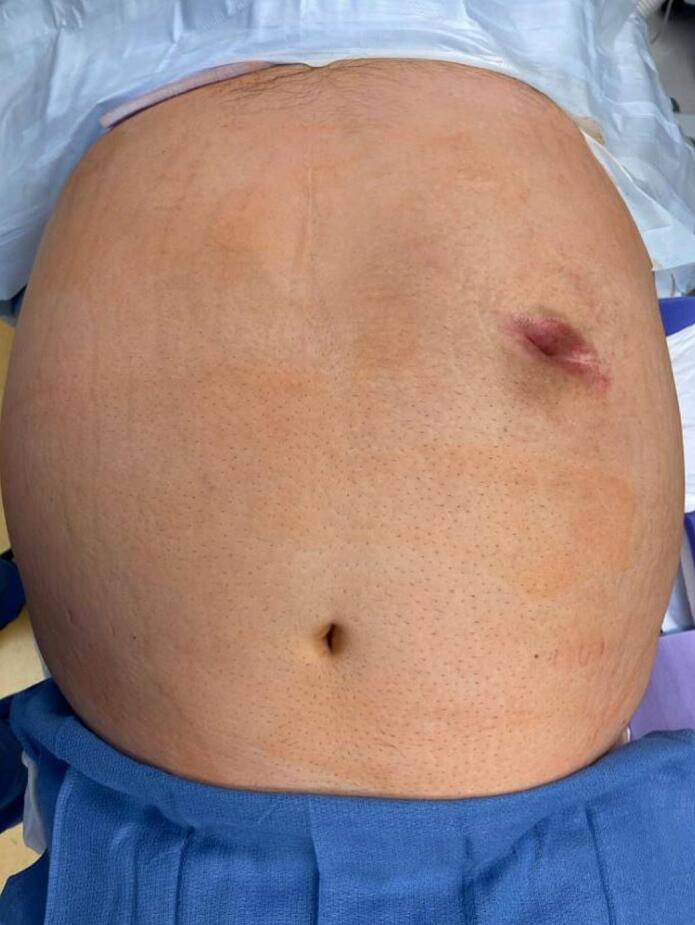
Intra-operative physical exam after initial draping and prep.

Outside imaging demonstrated a 5.2 cm fluid collection along the patient's prior gastrostomy tube site with passage of oral contrast [Fig. 1]. Conservative management including proton pump inhibitors and dietary changes were both tried and unsuccessful. Initial endoscopic evaluation by Gastroenterology demonstrated a large, internal fistula opening not amenable to clipping. Patient returned the following week and underwent endoscopic suturing of the internal fistula opening. Despite an initial improvement in symptoms, the patient noted mild discharge within the first few weeks after his procedure and full return of his symptoms after a few months. He was then referred to General Surgery for further evaluation. Initial examination demonstrated a large, external fistula opening within the left upper quadrant with expression of gastric contents [Fig. 2]. Patient was consented for a diagnostic laparoscopy and fistula takedown the following week.

**Fig. 3:**
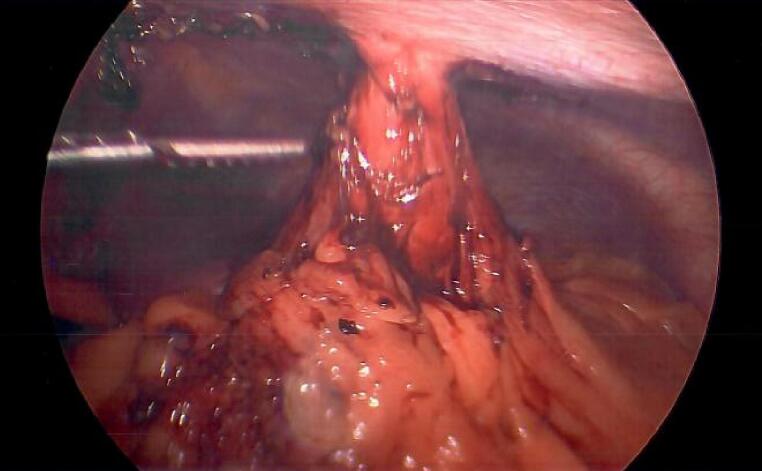
Intra-operative view of GC fistula using laparoscopy.

**Fig. 4:**
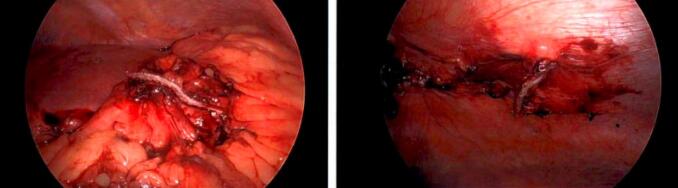
Staple line of resected GC fistula after using a green load Ethicon EndoGIA stapler. View of both stomach and abdominal wall. (For interpretation of the references to colour in this figure legend, the reader is referred to the web version of this article.)

In the operating room, the patient was laid supine with bilateral arms tucked. A total of three ports were placed within a diagonal line, triangulating towards the stomach. An obvious fistula tract was visualized between the anterior surface of the stomach and abdominal wall [Fig. 3]. Instrumentation of the fistulized tract aided in confirming and localizing the true tract amongst adhesions. The base of the fistula at the level of the stomach was transected using a green load stapler [Fig. 4]. Any remaining tract was then excised extracorporeally through an elliptical skin incision. Both dermis and skin were closed with suture. The patient was discharged home the same day and followed up in the clinic two weeks later with no complaints. Final pathology reported demonstrated a chronic fistula tract with no malignancy. He continues to deny any signs of recurrence after his six month follow up.

## 3 Discussion

When discussing persistent GCF, the primary source of risk assessment and management comes from the Pediatric Surgery literature. In children, the estimated rate of persistent GCF after gastrostomy tube removal varies between 2 and 40 % [2,3,11,12]. Within these studies, the only risk factor found to increase the odds of a patent GCF was the presence of a gastrostomy tube for greater than 6 months [1]. Janik et al. reported that only 10 % of children with a tube less than 8 months developed a persistent GCF, while those with a tube greater than 8 months had a rate up to 95 % [2]. A similar paper in adults by Currais et al. confirmed these results, and stated that no other patient factors were ever found to be statistically significant [1]. Our case varies from these studies in several ways. First, our patient reported a 41-year delay between the removal of his gastrostomy tube and formation of his GCF. To our knowledge, the only similar study comes from Beksac et al., which reports a 30 year delay in a pregnant female [9]. While previous studies focused on the persistent patency of a GCF, we demonstrate a rare case of a significantly delayed GCF. The unique aspect of our patient's timeline encourages further investigation into the cause of delayed GCF patency.

The second difference in our case comes from a lack of distinct risk factors. Our patient carried a gastrostomy tube for only 5 months, a month less than the risk factor detailed in prior studies [1]. The only obvious risk factor we identified was our patient's recent COVID pneumonia. We theorize that the immunosuppression and stress caused by our patient's COVID pneumonia led to the delayed patency of his GCF. Our hypothesis is supported in part by the findings of Beksac et al. [9] In that study, the only identifiable risk factor was the patient's pregnancy, which may have created a similar effect in immunosuppression and stress. While studies on persistent GCF patency do not see immunosuppression as a risk factor, we question whether the delayed patency of a GCF may carry a different set of risk factors. Given the rarity of our case, we hope that future cases may further delineate the true cause of delayed GCF patency.

Management of GCF fistulas remains a growing algorithm beginning with conservative measures such as acid suppression, pro-kinetics, and dietary changes [13]. Once conservative measures fail, a procedural approach begins. In Pediatrics, the gold standard for treatment is an open fistula takedown [12]. Adults have a wider range of options that include endoscopic closures, cauterizations, bedside fibrin plug, and combined therapies [1,[4], [5], [6], [7], [8],^14^]. While endoscopic and other non-surgical therapies provide significant benefits, studies have failed to demonstrate a unanimous advantage for any one therapy [1,4]. As such, failure in any of these methods should not delay immediate surgical referral for conclusive management. Our study demonstrated a minimally invasive approach that allowed the patient to receive definitive care in an outpatient setting. We believe that laparoscopic GCF takedown in the setting of a delayed GCF patency provides an excellent outcome with complete treatment in an outpatient setting.

## Submission declaration

The following article has not been published previously and is not under consideration for publication elsewhere.

## Guarantor

Brian Ward, MD.

## CRediT authorship contribution statement

Andrew Sabour, MD – Project administration, Conceptualization, Writing – original draft, Writing – review and editing.

Hannah Murawsky, BS – Writing – original draft.

Brian Ward, MD – Supervision, Writing – review and editing.

## Consent

Written informed consent was obtained from the patient for publication and any accompanying images. A copy of the written consent is available for review by the Editor-in-Chief of this journal on request.

## Ethical approval

Waived by the institution IRB due to the nature of being a case report.

## Sources of funding

None.

## Declaration of competing interest

None.

## References

[1] Currais P., Faias S., Francisco F., Sousa L., Gramacho J., Pereira A.D. (2021). Gastrocutaneous fistulas after PEG removal in adult cancer patients: frequency and treatment options. Surg. Endosc..

[2] Janik T.A., Hendrickson R.J., Janik J.S., Landholm A.E. (2004). Analysis of factors affecting the spontaneous closure of a gastrocutaneous fistula. J. Pediatr. Surg..

[3] Wu F.Y., Wu J.F., Ni Y.H. (2013). Long-term outcome after percutaneous endoscopic gastrostomy in children. Pediatr. Neonatol..

[4] Duddempudi S., Ghevariya V., Singh M., Krishnaiah M., Anand S. (2009). Treatment of persistently leaking post PEG tube gastrocutaneous fistula in elderly patients with combined electrochemical cautery and endoscopic clip placement. South. Med. J..

[5] Eskaros S., Ghevariya V., Krishnaiah M., Asarian A., Anand S. (2009). Percutaneous endoscopic suturing: an effective treatment for gastrocutaneous fistula. Gastrointest. Endosc..

[6] Hameed H., Kalim S., Khan Y.I. (2009). Closure of a nonhealing gastrocutanous fistula using argon plasma coagulation and endoscopic hemoclips. Can. J. Gastroenterol..

[7] Kothari T.H., Haber G., Sonpal N., Karanth N. (2012). The over-the-scope clip system--a novel technique for gastrocutaneous fistula closure: the first North American experience. Can. J. Gastroenterol..

[8] Teitelbaum J.E., Gorcey S.A., Fox V.L. (2005). Combined endoscopic cautery and clip closure of chronic gastrocutaneous fistulas. Gastrointest. Endosc..

[9] Beksac K., Konan A., Kaynaroglu V. (2017). Spontaneously closed gastrocutaneous fistula becomes symptomatic after 30 years with pregnancy. Clin. Exp. Obstet. Gynecol..

[10] Sohrabi C., Mathew G., Maria N. (2023). The SCARE 2023 guideline: updating consensus surgical CAse REport (SCARE) guidelines. Int. J. Surg..

[11] Deen O.J., Parisian K.R., Harris C., Kirby D.F. (2013). A novel procedure for gastrocutaneous fistula closure. J. Clin. Gastroenterol..

[12] Khirallah M.G., Bustangi N. (2020). Laparoscopic management of persistent gastrocutaneous fistula after feeding gastrostomy appliance removal in children. Ann. Pediatr. Surg..

[13] Masino E.E., Calderón Novoa F.M., Cano V., Wright F., Duro A. (2022). Gastrocutaneous fistula: Laparoscopic resolution. Rev. Gastroenterol. Mex..

[14] González-Ojeda A., Avalos-González J., Muciño-Hernández M.I. (2004). Fibrin glue as adjuvant treatment for gastrocutaneous fistula after gastrostomy tube removal. Endoscopy.

